# Supplementary material for: Design, Synthesis, and Use of Novel Photoaffinity Probes in Measuring the Serum Concentration of Glycogen Phosphorylase
Source: Molecules. 2019 Feb 22;24(4):798. doi: 10.3390/molecules24040798 (PMC6413153; doi:10.3390/molecules24040798)

Supporting Information

Design, Synthesis, and Use of Novel Photoaffinity Probes in Measuring the Serum Concentration of Glycogen Phosphorylase

Zhang Yuchao, Wang Youde, Yan Zhiwei, Song Chengjun, Miao Guangxin, Zhang Liying.

Contents

Figure S1: Copy of ^{1}H NMR and ^{13}C NMR of 5 3
Figure S2: Copy of ^{1}H NMR and ^{13}C NMR of 6 4
Figure S3: Copy of ^{1}H NMR and ^{13}C NMR of 7 5
Figure S4: Copy of ^{1}H NMR and ^{13}C NMR of 9 6
Figure S5: Copy of ^{1}H NMR and ^{13}C NMR of 11 7
Figure S6: Copy of ^{1}H NMR and ^{13}C NMR of 12 8
Figure S7: Copy of ^{1}H NMR and ^{13}C NMR of 13 9
Figure S8: Copy of ^{1}H NMR and ^{13}C NMR of 20 10
Figure S9: Copy of ^{1}H NMR and ^{13}C NMR of 1 11
Figure S10: Copy of ^{1}H NMR and ^{13}C NMR of 2 12
Figure S11: Copy of ^{1}H NMR and ^{13}C NMR of 3 13

^{1}H NMR spectra of compound **5**

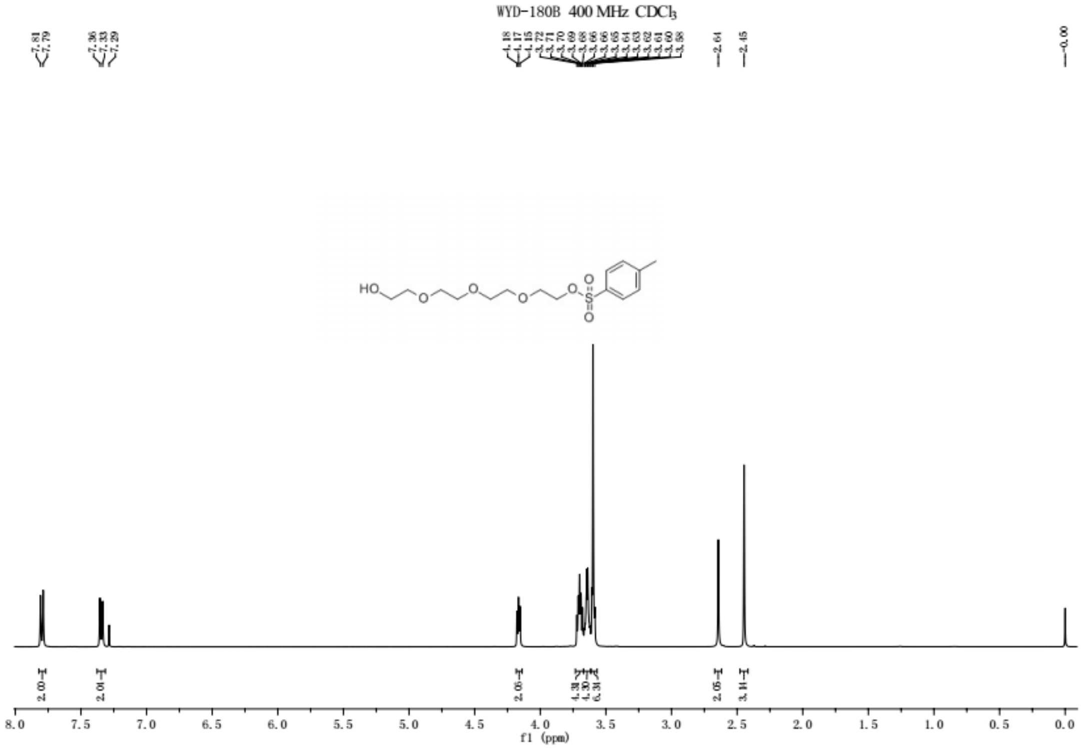

^{13}C NMR spectra of compound **5**

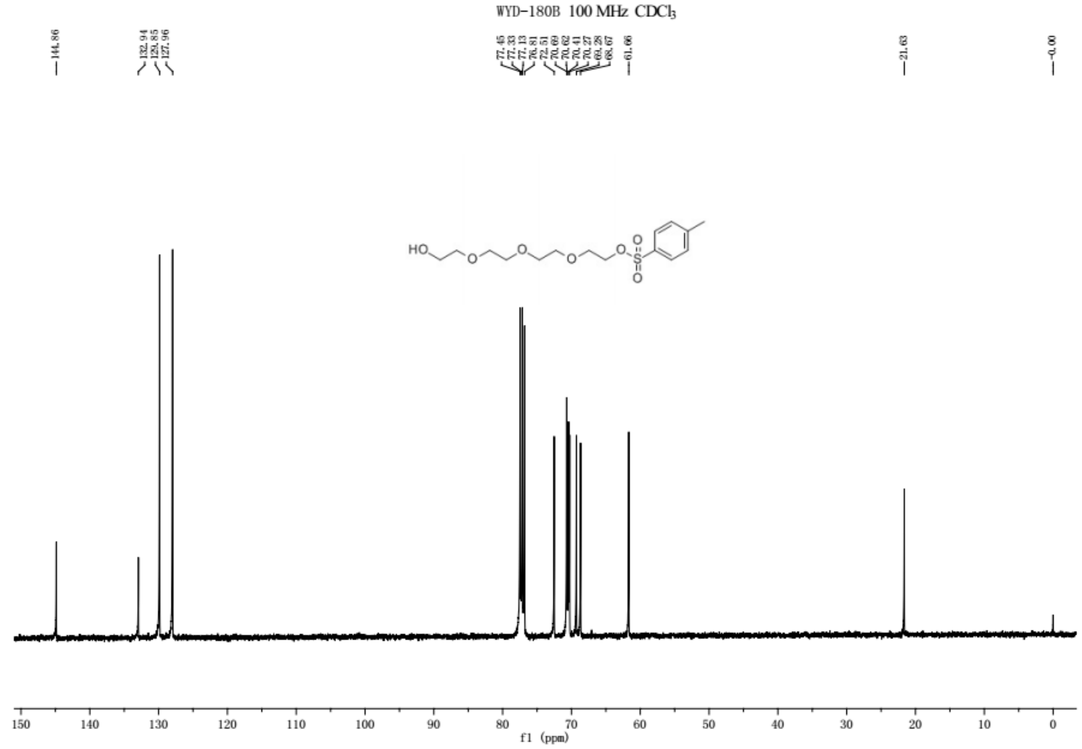

^{1}H NMR spectra of compound **6**

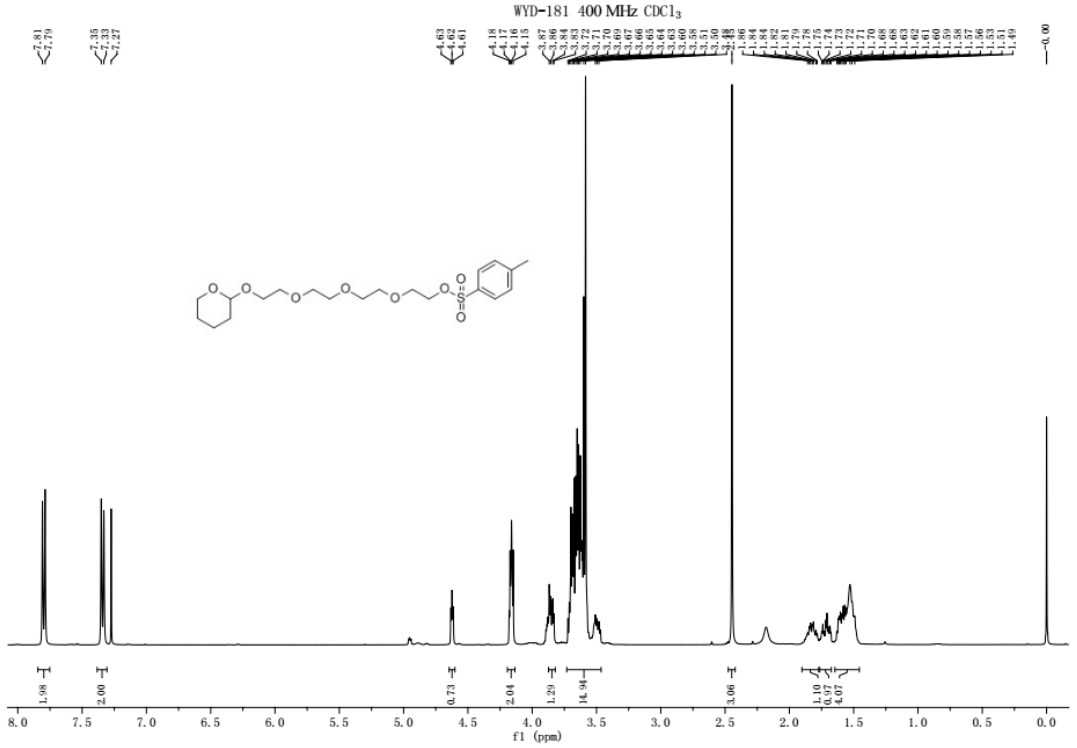

^{13}C NMR spectra of compound **6**

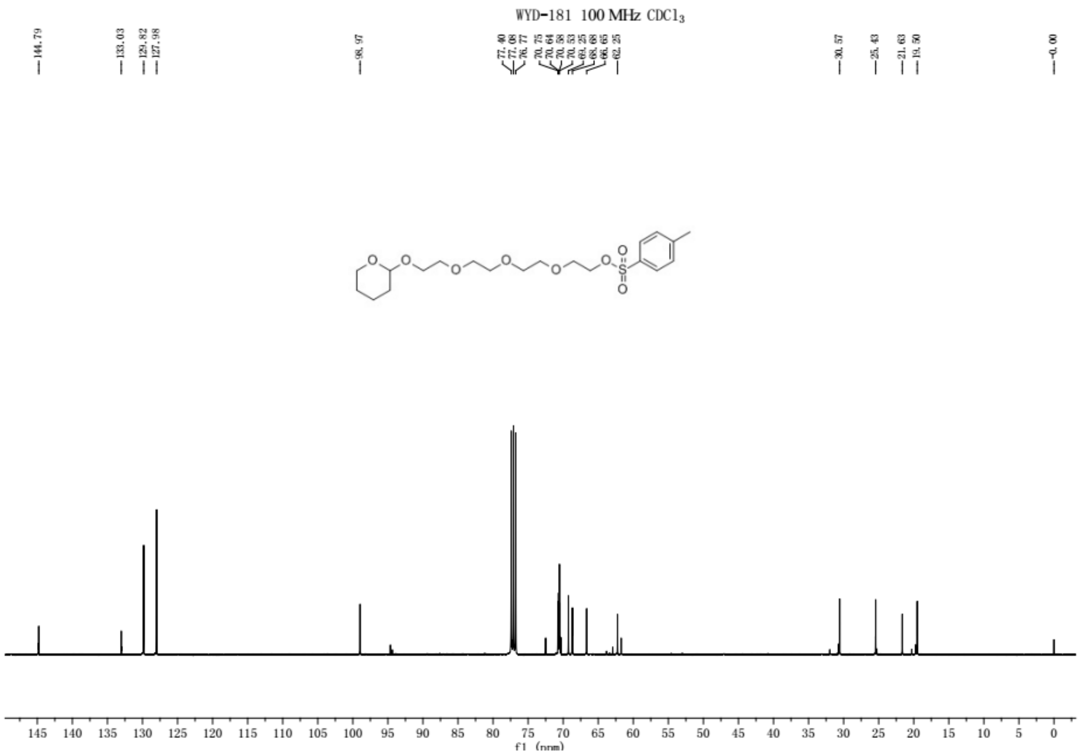

^{1}H NMR spectra of compound **7**

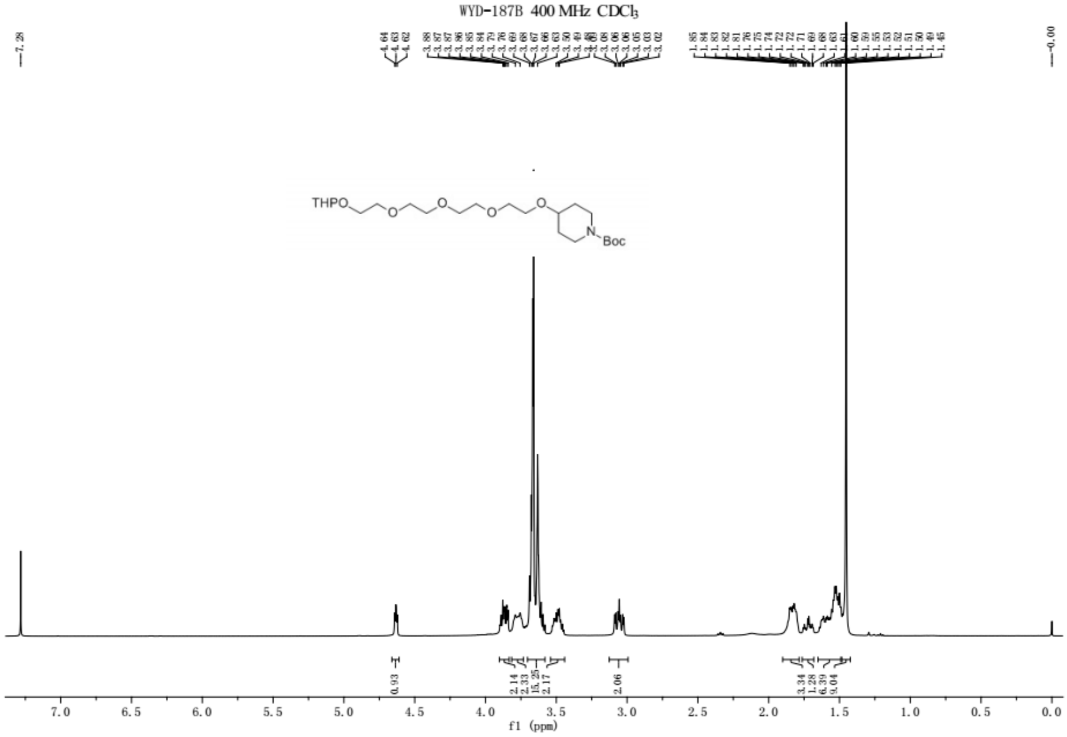

^{13}C NMR spectra of compound **7**

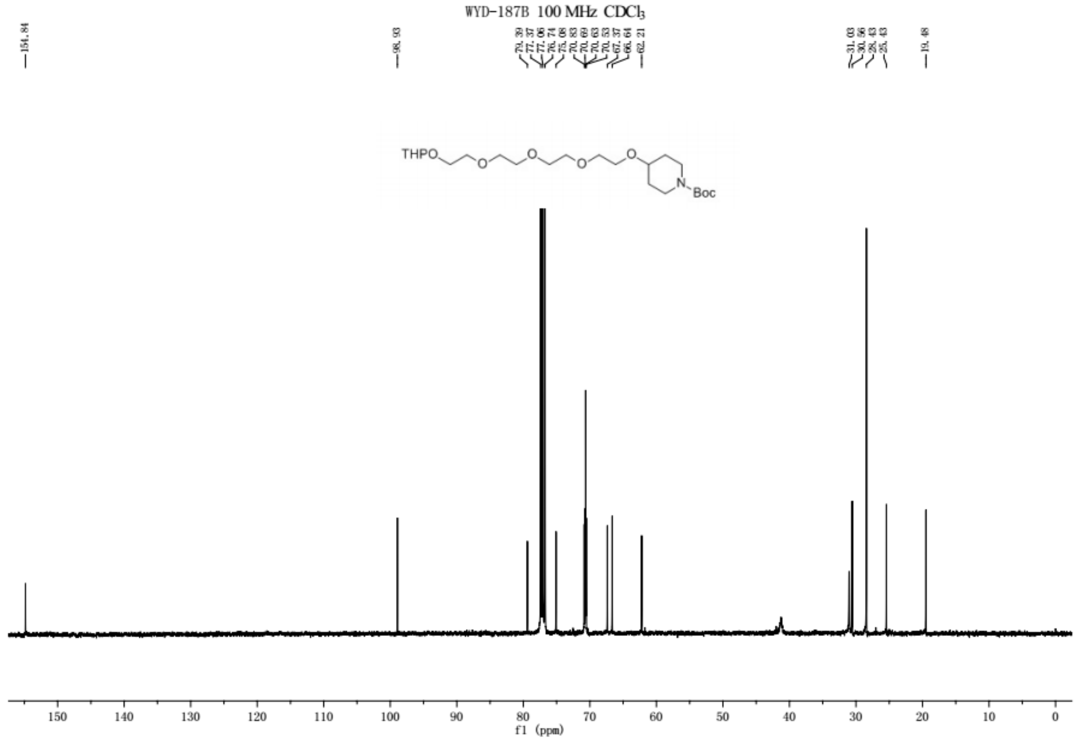

^{1}H NMR spectra of compound **9**

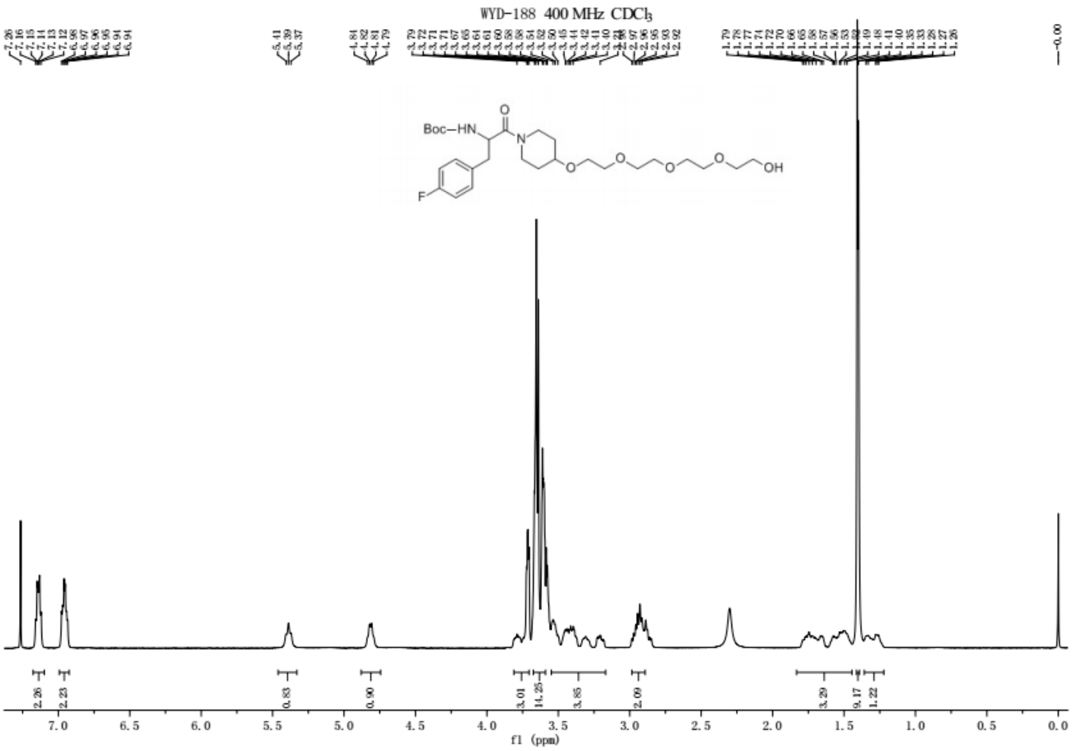

^{13}C NMR spectra of compound **9**

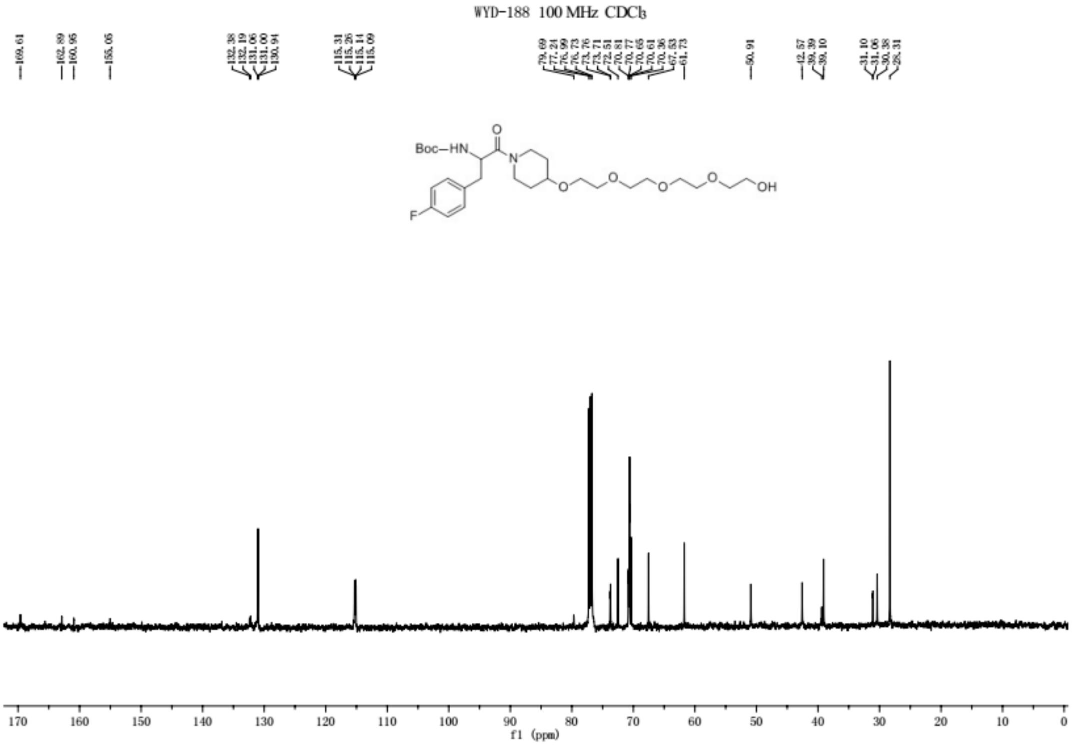

^{1}H NMR spectra of compound **11**

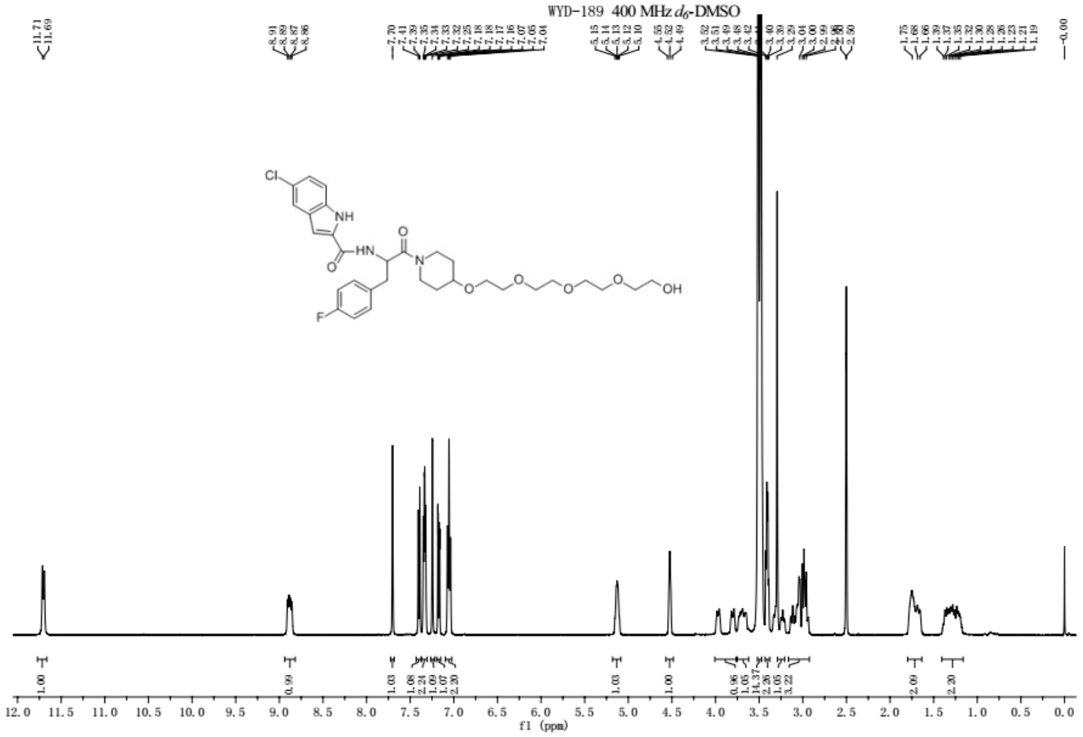

^{13}C NMR spectra of compound **11**

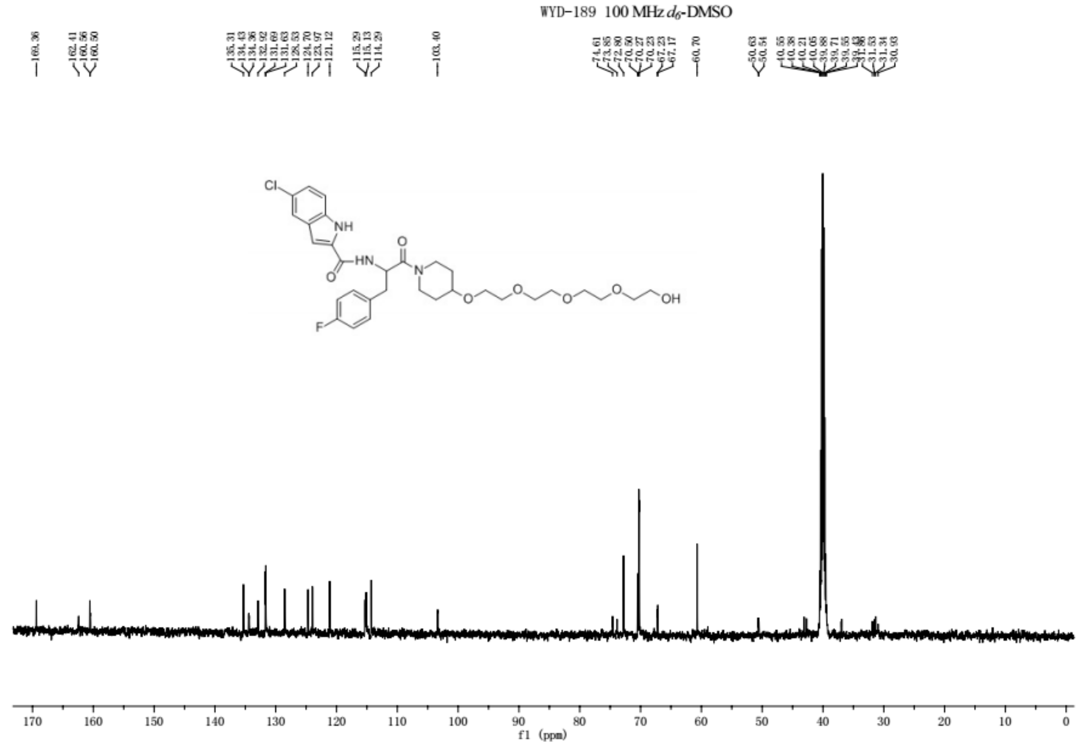

^{1}H NMR spectra of compound **12**

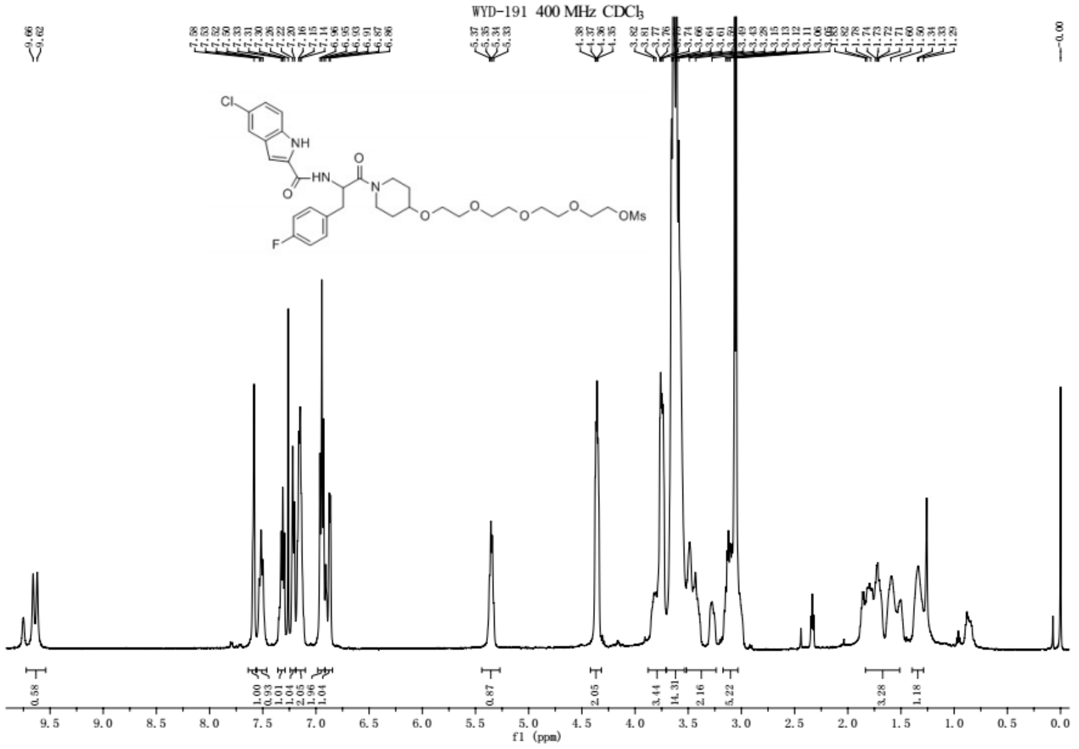

^{13}C NMR spectra of compound **12**

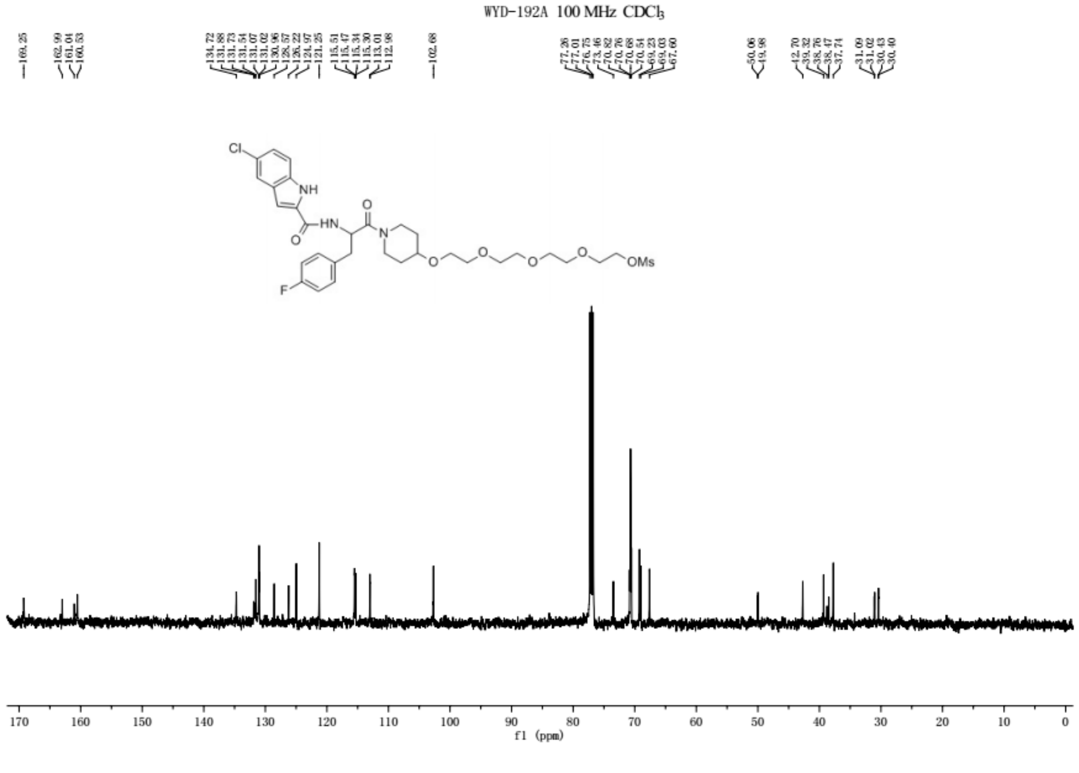

^{1}H NMR spectra of compound **13**

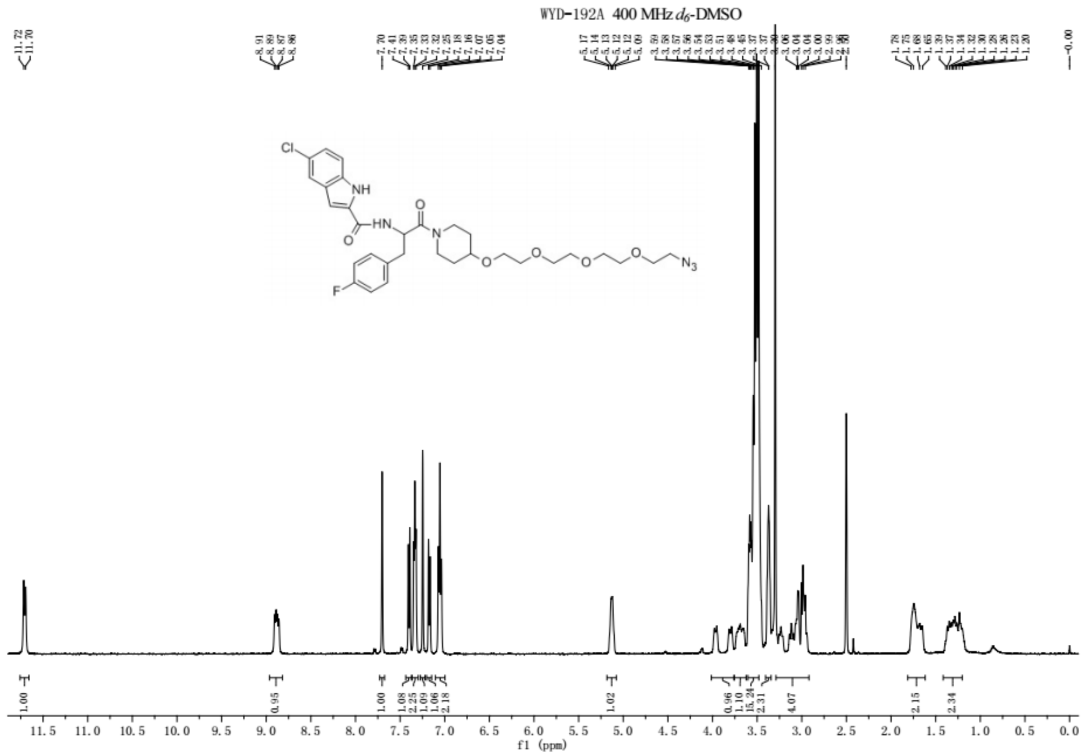

^{13}C NMR spectra of compound **13**

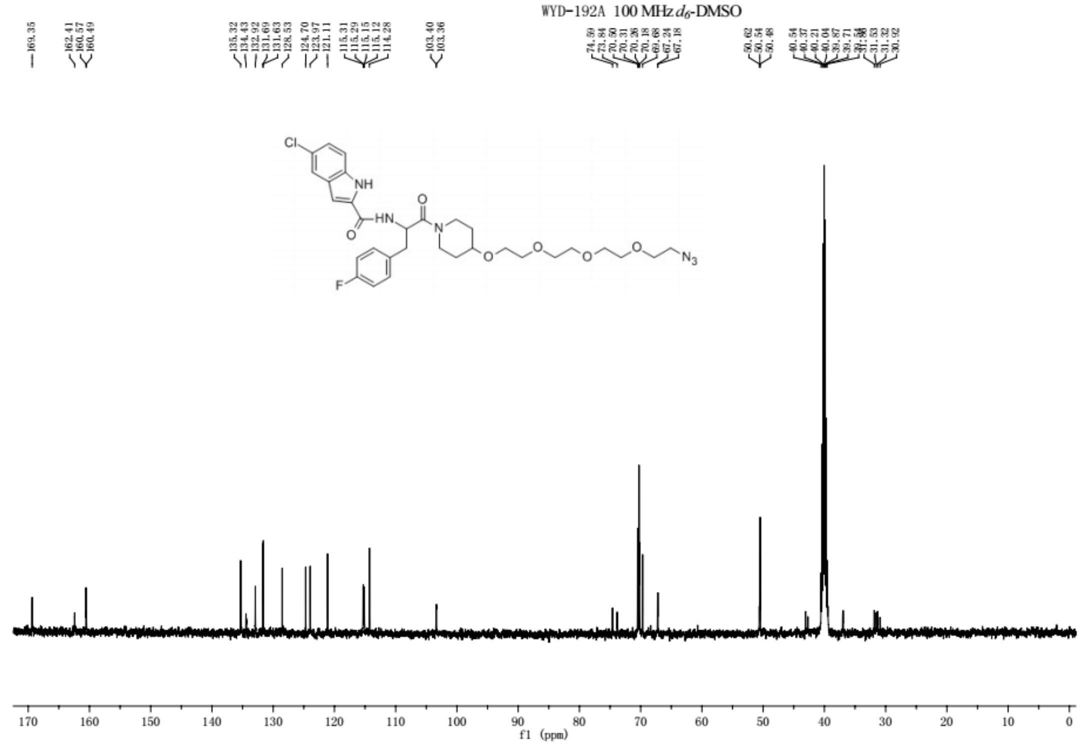

^{1}H NMR spectra of compound **20**

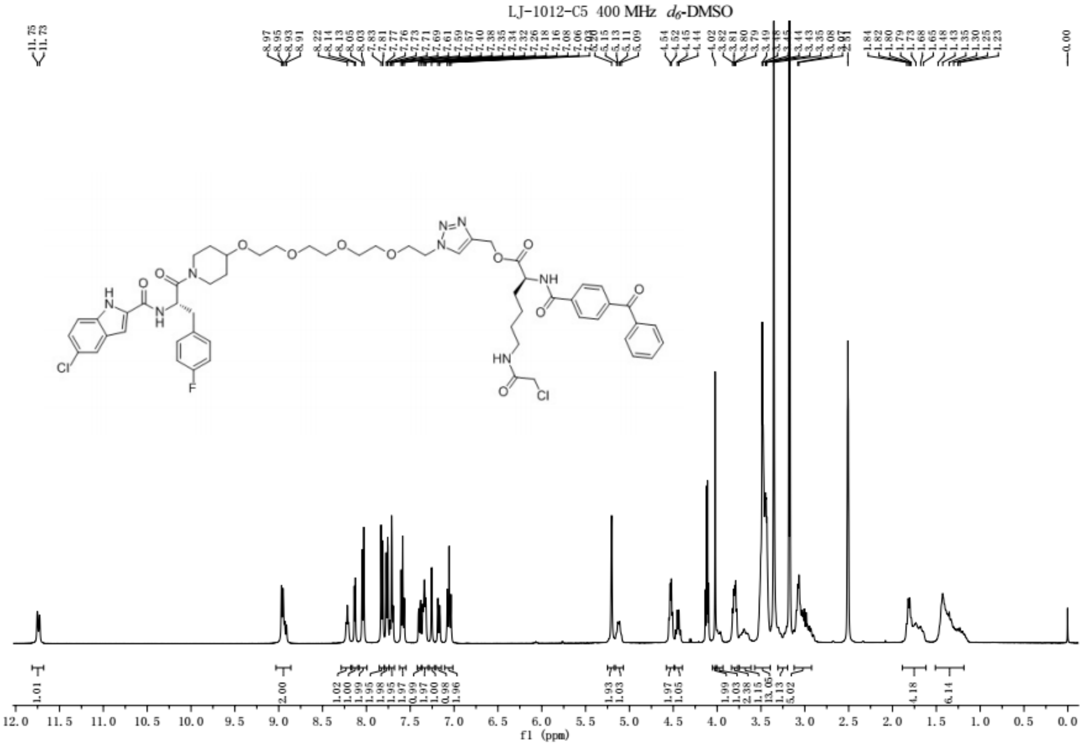

^{13}C NMR spectra of compound **20**

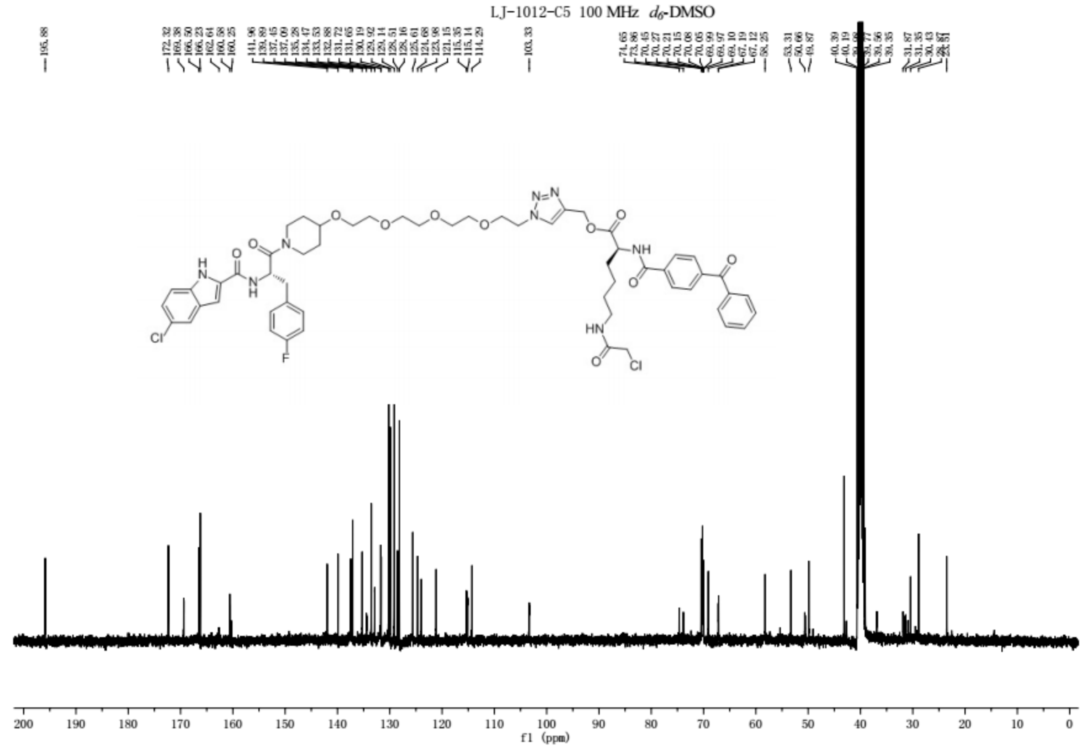

^{1}H NMR spectra of compound **1**

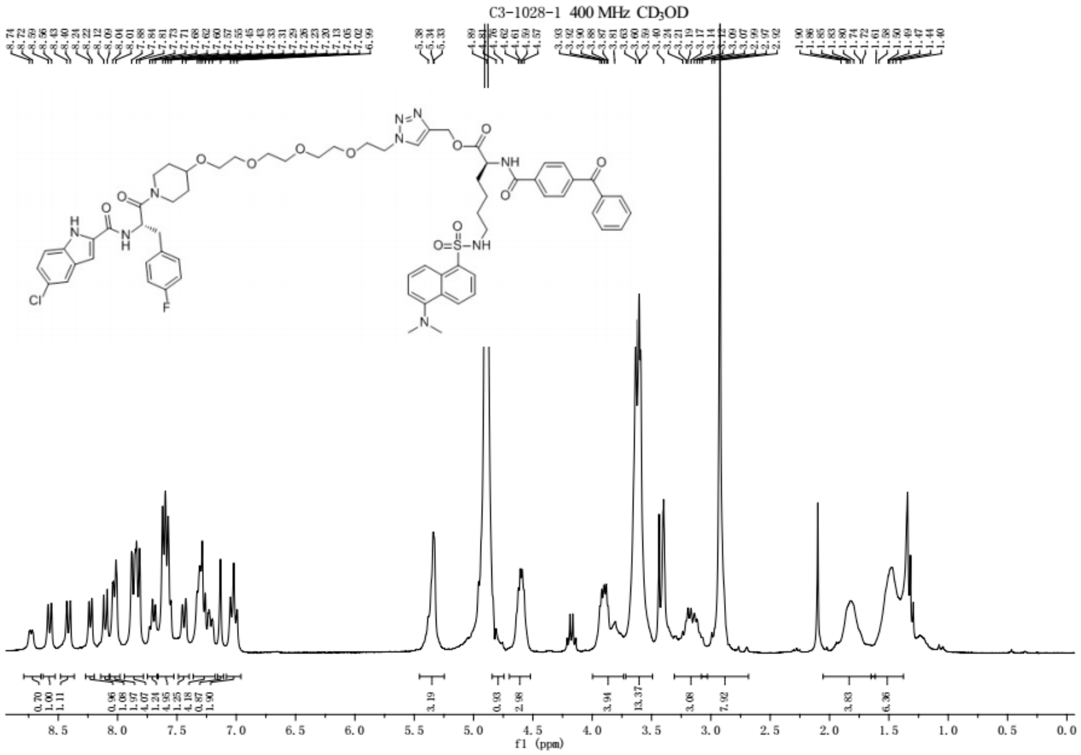

^{13}C NMR spectra of compound **1**

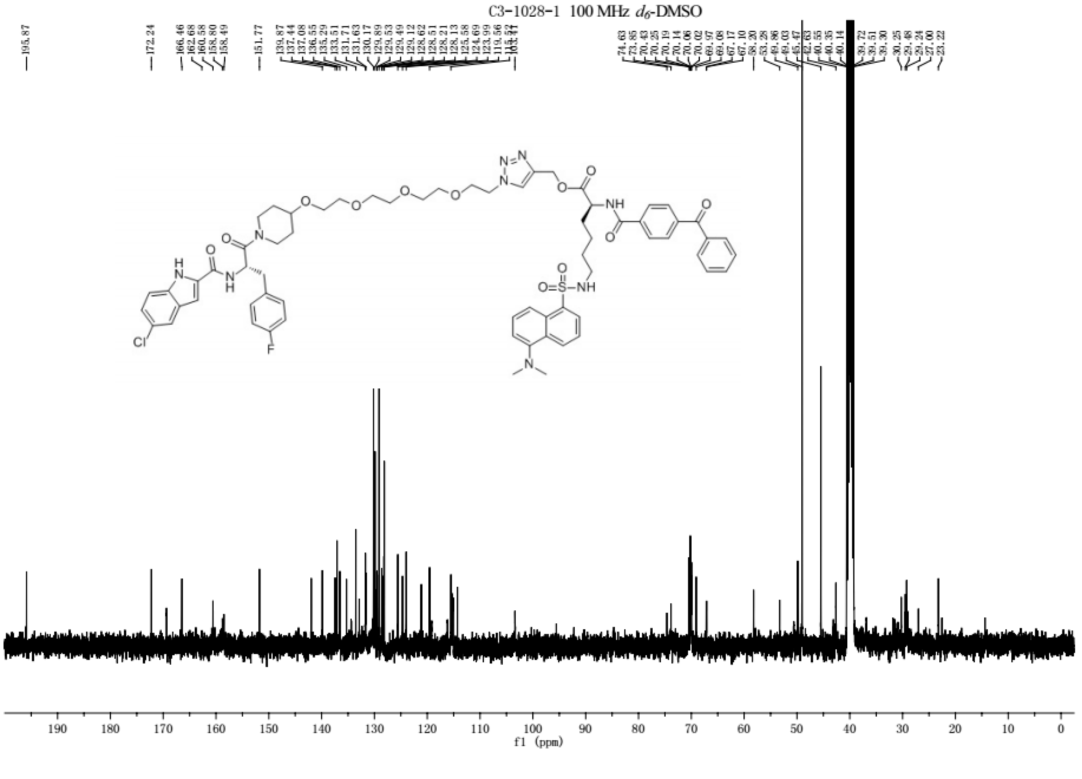

^{1}H NMR spectra of compound **2**

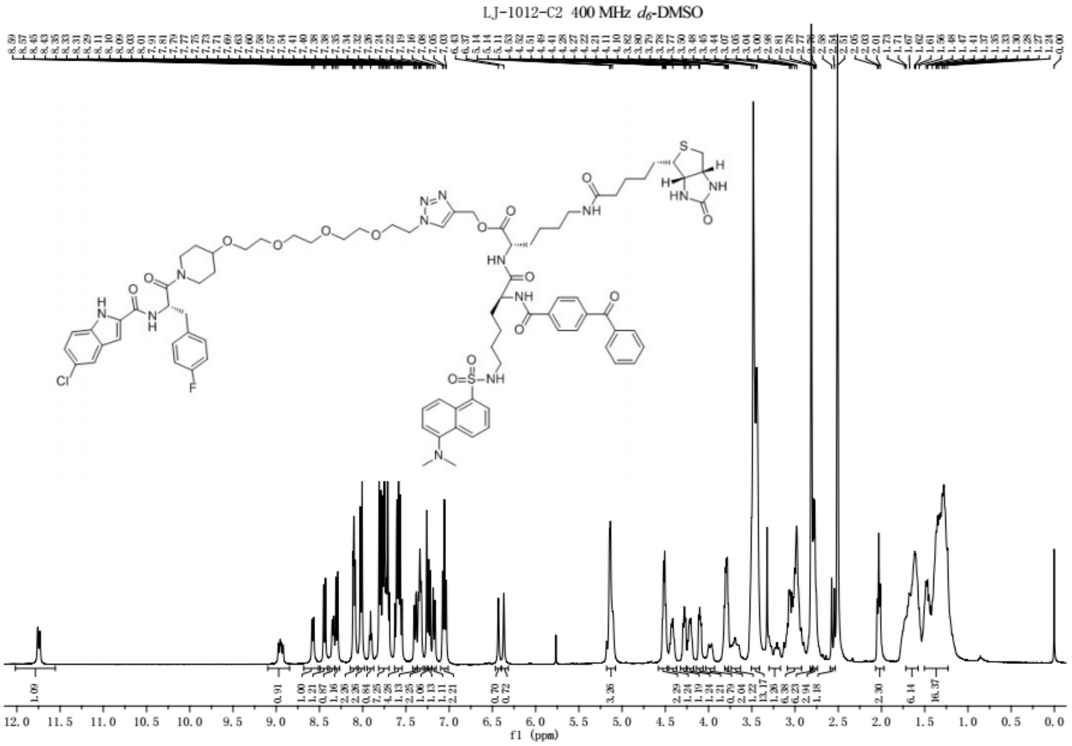

^{13}C NMR spectra of compound **2**

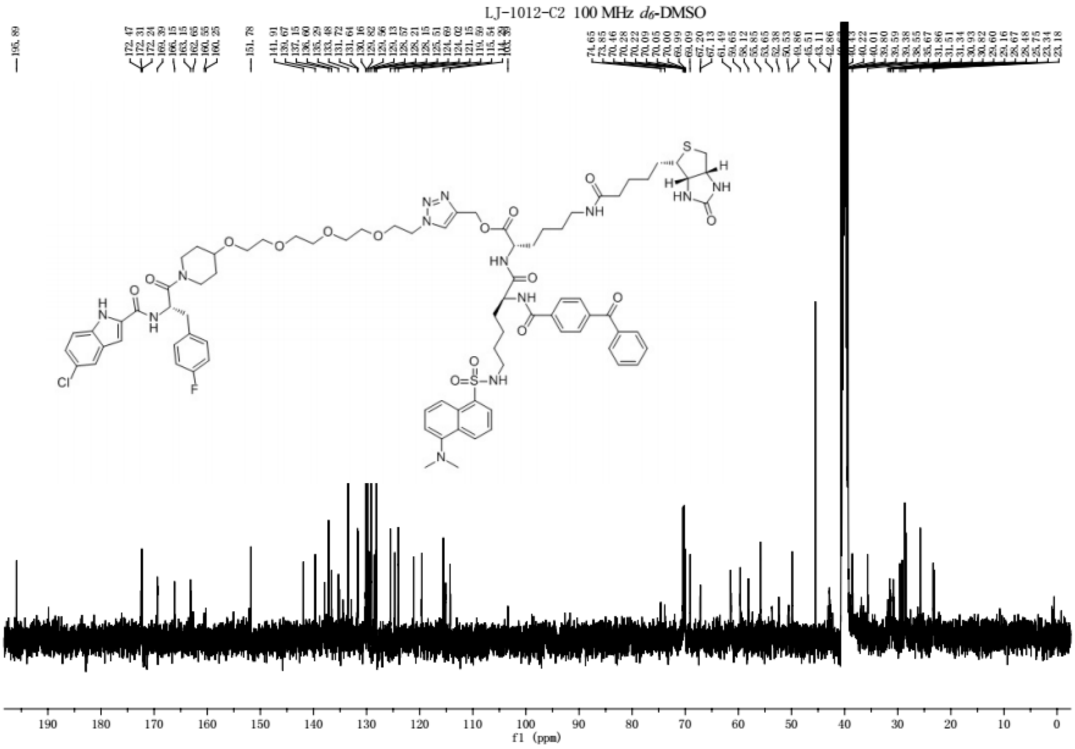

^{1}H NMR spectra of compound **3**

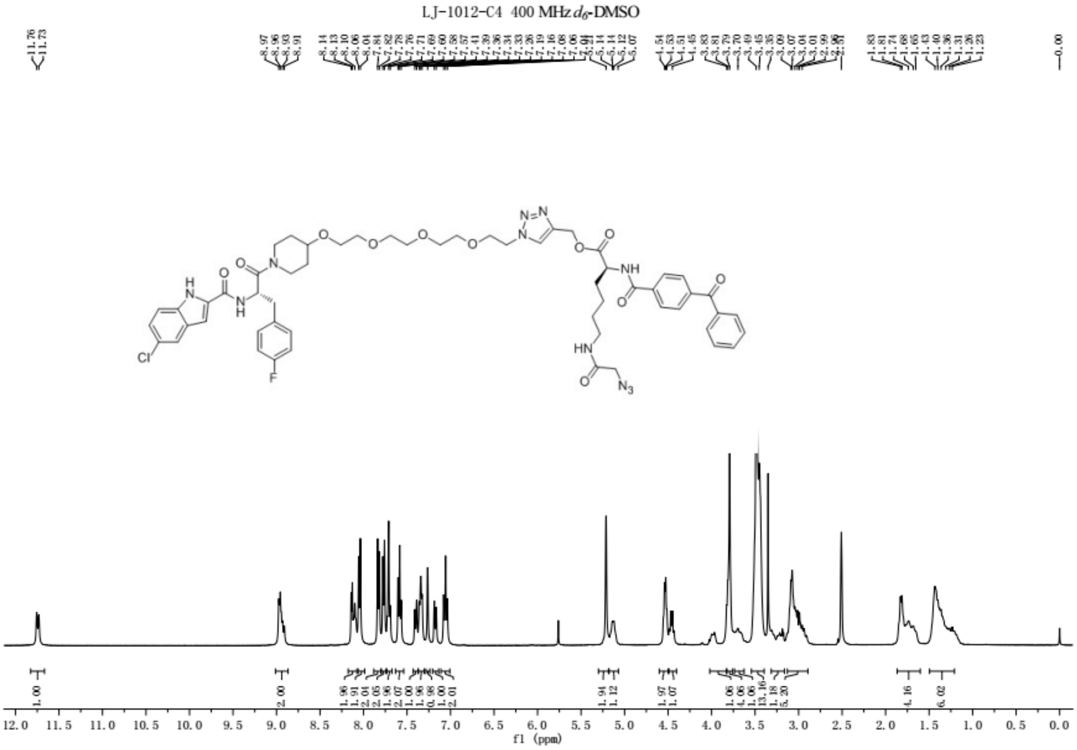

^{13}C NMR spectra of compound **3**

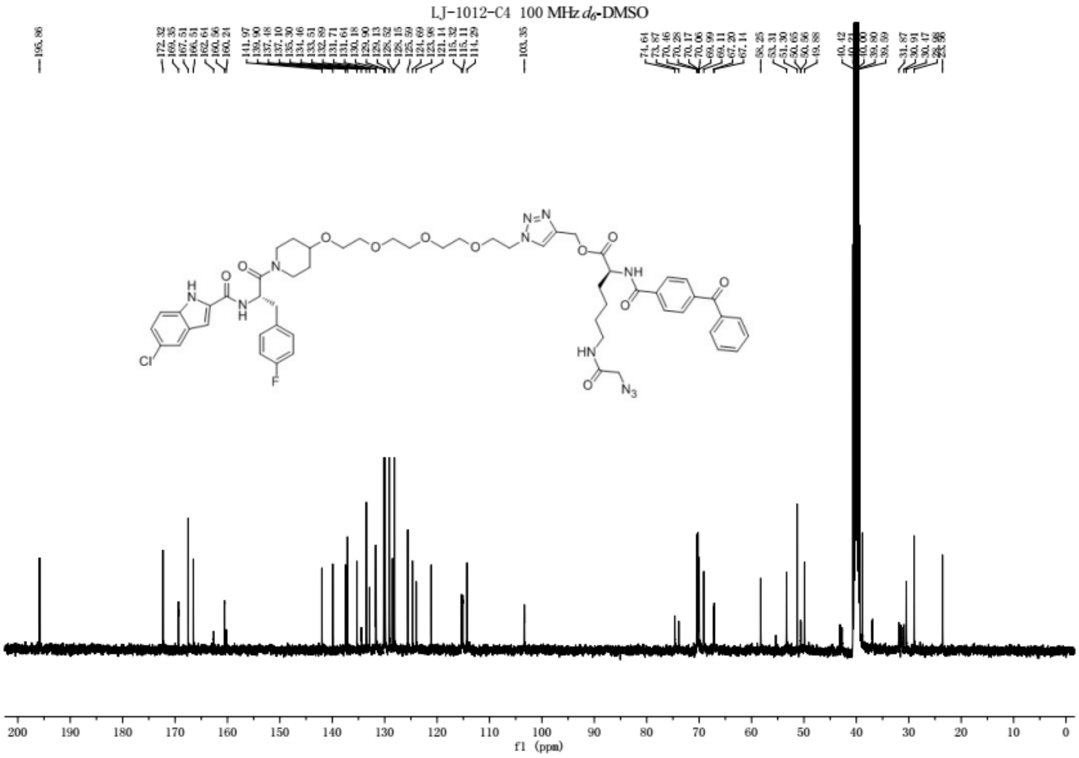

Supplement: Supplementary file 1 [file molecules-24-00798-s001.pdf]
